# Differentiating Malignant from Tubercular Pleural Effusion by Cancer Ratio Plus (Cancer Ratio: Pleural Lymphocyte Count)

**DOI:** 10.1155/2016/7348239

**Published:** 2016-12-14

**Authors:** Akash Verma, Rucha S. Dagaonkar, Dominic Marshall, John Abisheganaden, R. W. Light

**Affiliations:** ^1^Department of Respiratory and Critical Care Medicine, Tan Tock Seng Hospital, Singapore 308433; ^2^Department of Medicine, Imperial College London, London SW7 2AZ, UK; ^3^Pulmonary Disease Program, Vanderbilt University, 1161 21st Ave. South, Nashville, TN 37232, USA

## Abstract

*Background*. We performed prospective validation of the cancer ratio (serum LDH : pleural ADA ratio), previously reported as predictive of malignant effusion retrospectively, and assessed the effect of combining it with “pleural lymphocyte count” in diagnosing malignant pleural effusion (MPE).* Methods*. Prospective cohort study of patients hospitalized with lymphocyte predominant exudative pleural effusion in 2015.* Results*. 118 patients, 84 (71.2%) having MPE and 34 (28.8%) having tuberculous pleural effusion (TPE), were analysed. In multivariate logistic regression analysis, cancer ratio, serum LDH : pleural fluid lymphocyte count ratio, and “cancer ratio plus” (ratio of cancer ratio and pleural fluid lymphocyte count) correlated positively with MPE. The sensitivity and specificity of cancer ratio, ratio of serum LDH : pleural fluid lymphocyte count, and “cancer ratio plus” were 0.95 (95% CI 0.87–0.98) and 0.85 (95% CI 0.68–0.94), 0.63 (95% CI 0.51–0.73) and 0.85 (95% CI 0.68–0.94), and 97.6 (95% CI 0.90–0.99) and 94.1 (95% CI 0.78–0.98) at the cut-off level of >20, >800, and >30, respectively.* Conclusion*. Without incurring any additional cost, or requiring additional test, effort, or time, cancer ratio maintained and “cancer ratio plus” improved the specificity of cancer ratio in identifying MPE in the prospective cohort.

## 1. Introduction

The initial work-up of pleural effusion entails biochemical, microbiological, and cytological examination of the pleural fluid [1]. Biochemical tests routinely and universally performed in clinical practice for investigating pleural effusion are serum lactate dehydrogenase (LDH) and protein, pleural LDH, protein, differential cell count, pH, glucose, and adenosine deaminase (ADA) [2].

Tuberculous pleural effusion (TPE), malignant pleural effusion (MPE), and parapneumonic pleural effusion are the most common aetiologies of an exudative pleural effusion in clinical practice [3]. In this context, among routinely performed pleural fluid analyses, neutrophilic predominance is indicative of a parapneumonic pleural effusion, and a raised ADA level is highly suggestive (specificity of 92%) for TB, but to date, no test is specific to “rule-in” MPE [4, 5]. Given the sinister nature of this pathology, low diagnostic yield of pleural fluid cytology (~60%), and the invasive nature of closed or thoracoscopic pleural biopsy, this is a significant limitation for routinely performed biochemical tests [6–8]. This inability presents itself both as a challenge, and an opportunity for improvement. In recent years, several more advanced assays have been developed to diagnose malignancy in a patient presenting with pleural effusion. Examples include measurement of tumour markers CEA, CA15-3, CA125, and cyfra 21-1 in pleural fluid and protein microarray technologies to differentiate malignant from TB effusion [9, 10]. Although these new techniques have potential, their use has not entered mainstream practice. In addition, they carry cost implications and lack availability in many centres.

Among the routinely performed biochemical tests for investigating pleural effusion, serum lactate dehydrogenase (LDH), pleural ADA, and pleural lymphocyte count change in reciprocal manner in patients with MPE and TPE. Serum LDH is raised in MPE whereas pleural ADA and pleural fluid lymphocyte count remain comparatively low. Conversely, serum LDH is low in TPE whereas pleural ADA and pleural fluid lymphocyte count are raised. This reciprocal change presents an opportunity to combine these test results developing a ratio with the diagnostic power to differentiate MPE from TPE in a cost effective, timely, generalizable, and universally applicable manner. Such a marker not only may provide an early signal toward malignant nature of pleural effusion, but can potentially serve as a “forewarning” for patients with negative cytology who are subsequently found to have MPE. Our previous report of a retrospective analysis demonstrated that a “cancer ratio” (serum LDH : pleural ADA ratio) yielded sensitivity and specificity of 0.98 and 0.94, respectively, at the cut-off level of >20 for identifying MPE [11].

In this study, our primary objective was to prospectively validate the use of our previously described “cancer ratio” for its association with MPE and assess its utility to differentiate MPE from TPE. Secondary objectives included exploring the utility of combining pleural lymphocyte counts to generate a “cancer plus ratio” in identifying MPE.

## 2. Material and Methods

### 2.1. Data Collection

Patients hospitalized in Tan Tock Seng Hospital consecutively for lymphocytic predominant exudative pleural effusion during the year 2015 were studied. We excluded patients with transudative effusion and neutrophilic predominant exudative pleural effusion. Data was collected on age, gender, serum LDH, serum protein, pleural LDH, pleural protein, pleural fluid differential cell count, pleural fluid ADA, cytology, pleural fluid microbiology results, and pleural biopsy results.

### 2.2. Ratios

We calculated and analysed three ratios:

1. The ratio between serum LDH and pleural ADA: this was called “cancer ratio” as per our previous publication [11]. This was calculated for prospective validation of our previous retrospectively published findings.
2. The ratio of cancer ratio to the percentage of differential pleural lymphocyte count: this was called “cancer ratio plus.” It was calculated to assess the effect of combining pleural lymphocyte count with the cancer ratio on the accuracy of identifying MPE.
3. The ratio of serum LDH and differential pleural lymphocyte count: as we did with cancer ratio plus, this ratio was calculated to assess the effect of combining pleural lymphocyte count with the serum LDH on the accuracy of identifying MPE.

### 2.3. Statistical Analysis

We used software (SPSS, version 17; SPSS, Chicago, IL) for all statistical analyses. The results were compared using a Wilcoxon two-sample test or Fisher exact test.* P* values were two sided and considered indicative of a significant difference if <0.05. Multivariate logistic regression analysis was done along with receiver operating curve (ROC) analysis and calculation of area under the curve (AUC) values.

## 3. Results

A total of 118 patients with lymphocytic predominant exudative pleural effusion were analysed: 84 (71.2%) had MPE and 34 (28.8%) had TPE. Among those with MPE, the aetiology of malignancy was as follows: primary lung cancer (*n* = 82), mesothelioma (*n* = 1), and lymphoma (*n* = 1). Patient characteristics and laboratory values are described in Table 1. For those in whom pleural fluid cytology was negative (*n* = 9), patients underwent EBUS-TBNA (*n* = 2), pleural biopsy (*n* = 1), tongue biopsy (*n* = 1), ETT aspirate (*n* = 1), and lung biopsy (*n* = 4), for the confirmation of the diagnosis.

Univariate analysis showed pleural fluid differential lymphocyte count to be significantly lower and cancer ratio significantly higher in MPE as compared to TPE, Table 1.

When pleural fluid lymphocyte count was combined with serum LDH as serum LDH : pleural fluid lymphocyte count ratio, and cancer ratio as ratio of cancer ratio and pleural fluid lymphocyte count (cancer ratio plus), a further discriminating effect between malignant and TB pleural effusion was seen. In multivariate logistic regression analysis, cancer ratio, serum LDH : pleural fluid lymphocyte count ratio, and “cancer ratio plus” maintained significance as positive predictors of MPE, Table 2.

ROC analysis was done to derive cut-off levels providing best trade-off between sensitivity and specificity for each of the ratios that maintained significance in the multivariate logistic regression analysis.

### 3.1. Cancer Ratio

**Figure 1:**
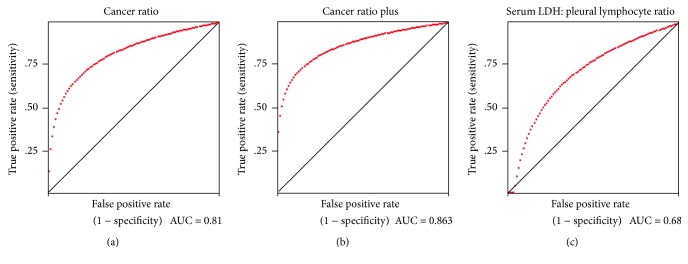
(a) ROC curve for various cut-off levels of cancer ratio in differentiating between malignant pleural effusions from TB pleural effusion. AUC of 0.81 suggests good accuracy of this test. (b) ROC curve for various cut-off levels of cancer ratio plus (cancer ratio: pleural fluid lymphocyte count) in differentiating between malignant pleural effusion from TB pleural effusion. AUC of 0.86 suggests good accuracy of this test. (c) ROC curve for various cut-off levels of serum LDH : pleural lymphocyte count ratio in differentiating between malignant pleural effusion from TB pleural effusion. AUC of 0.68 suggests poor accuracy of this test.

At cut-off level of >20, the sensitivity and specificity of “cancer ratio” were 0.95 (95% CI 0.87–0.98) and 0.85 (95% CI 0.68–0.94), respectively. The positive likelihood ratio (PLR) value was 16, while the negative likelihood ratio (NLR) at this cut-off was found to be 0.13, Table 3. Area under the curve (AUC) was 0.81 (Figure 1).

### 3.2. Cut-Off Level for Cancer Ratio Plus (Cancer Ratio: Pleural Fluid Lymphocyte Count)

At cut-off level of >30, the sensitivity and specificity of “cancer ratio plus” were 0.97 (95% CI 0.90–0.99) and 0.94 (95% CI 0.78–0.98), respectively. The positive likelihood ratio (PLR) value was 41, while the negative likelihood ratio (NLR) at this cut-off was found to be 0.06. AUC was 0.86. At cut-off level of >20, the sensitivity was 1.0 (95% CI 0.94–1.0), Table 4.

### 3.3. Cut-Off Level for Serum LDH : Pleural Lymphocyte Count Ratio

In the case of serum LDH : pleural lymphocyte count ratio, the optimum sensitivity and specificity was found at the cut-off level of ≥800. The sensitivity was 0.63 (95% CI 0.51–0.73) and specificity was 0.85 (95% CI 0.68–0.94). These values were lower than the sensitivity and specificity of “cancer ratio” and “cancer ratio plus.” Additionally, PLR was low (10.6), and NLR was high (1.06) at this cut-off level indicating unreliability of this test. Area under the curve on the ROC curve was 0.68 again indicating serum LDH : pleural lymphocyte count ratio to be a poorer test in discriminating MPE from TPE (Figure 1).

## 4. Discussion

In this prospective cohort analysis our “cancer ratio” was effective in identifying MPE, validating previous findings. In addition, we report further enhancement in accuracy of “cancer ratio” when combined with pleural fluid lymphocyte count (cancer ratio plus). A cut-off level of the “cancer ratio plus” of >30 was highly predictive of MPE in patients with lymphocyte predominant exudative pleural effusion, with both high sensitivity (0.97) and specificity (0.94). The positive likelihood ratio was 41, while the negative likelihood ratio was 0.06.

### 4.1. Cancer Ratio

The ROC-derived cut-off level of >20 for cancer ratio in our prospective cohort yielded sensitivity and specificity of 0.95 and 0.85 and PLR and NLR of 16 and 0.13, respectively. These findings were slightly lower than the previous results of our retrospective study on cancer ratio where this cut-off level allowed distinction of MPE from nonmalignant pleural effusion with the sensitivity and specificity of 0.98 and 0.94 and PLR and NLR of 32.6 and 0.03, respectively [11].

The reason for the lower sensitivity and specificity of cancer ratio in the prospective cohort was not apparent. However, it can be speculated that the lower median serum LDH level in the prospective cohort as compared to retrospective cohort may be responsible. In the retrospective study, blood samples on which LDH level was tested were haemolysed in several patients. This was reported as one of the limitations of the study [11]. Haemolysis can falsely elevate serum LDH levels. This elevation may have magnified the difference in serum LDH levels between the malignant and nonmalignant groups. In contrast, we excluded serum LDH of haemolysed samples in our prospective cohort. This could be the reason why, although the trend of higher serum LDH levels in patients with MPE as compared to TPE was seen, it did not reach statistical significance.

### 4.2. Pleural Lymphocyte Count

The median lymphocyte count percentage in our cohort was higher in TPE than MPE (86% versus 61%, *P* < 0.007), consistent with previous reports. High percentages of lymphocytes in the pleural fluid have been shown to be associated with TPE. 67% percent of patients with TPE in one study were reported to have pleural lymphocyte percentage of >95% [12]. In another study of 245 patients with TPE, >50% of leukocytes in pleural fluid were lymphocytes with mean ± SD of 77 ± 19.9 and median (range) of 80.5 (2–100%) [13]. In a larger study of 382 patients with TPE, median lymphocyte percentage of total cells was 84% [14]. Several other studies have described lymphocyte predominance in 60–90% of cases of TPE [15–17]. Only exceptionally (in ~5%) lymphocyte count of <50% may occur [18]. Thus, when 80% lymphocyte is chosen as the reference level, TPE is by far the most frequent cause of pleural lymphocytosis [19]. The proposed mechanism of TPE is the interaction between* Mycobacterium tuberculosis* and the human immune system, causing hypersensitivity reaction to mycobacterial proteins in the pleura [20]. This finding formed the basis of the design of our study owing to the reciprocal change seen between pleural lymphocyte count and serum LDH and pleural ADA in MPE. Although neutrophil predominance can be seen in MPE, the incidence is low at around 8% [21]. Correspondingly, 9.5% of patients had neutrophil predominance in our cohort of MPE.

### 4.3. Cancer Ratio Plus (Cancer Ratio: Pleural Fluid Lymphocyte Ratio)

The idea of combining the biomarkers to improve accuracy of tests in diagnosing pleural effusion is not novel. Diacon et al. described the improvement in specificity of ADA to 100% when combined with pleural lymphocyte : neutrophil ratio (L : N ratio), as compared to 95% when used alone for diagnosing TPE [22]. Similarly Burgess et al. demonstrated improvement in specificity of ADA from 81% to 95% by combining it with L : N ratio for diagnosing TPE [23].

While the ROC-derived cut-off level of “cancer ratio” allowed distinction of MPE from TPE with sensitivity and specificity of 0.95 and 0.85, the cut-off level of “cancer ratio plus” of >30 improved the sensitivity and specificity to 0.97 and 0.94, respectively. The PLR at this cut-off level was 41, while the NLR was found to be 0.06. A PLR value of 41 suggests that patients with cancer have about 41-fold higher chance of having “cancer ratio plus” of >30 compared with patients without cancer. This high probability would be considered high enough to consider an effusion very likely to be malignant. In contrast, NLR at this cut-off was found to be 0.06 which suggests that if the “cancer ratio plus” is <30, the probability that this patient has cancer is 6%, which is low enough to make the diagnosis of cancer highly unlikely.

### 4.4. Serum LDH : Pleural Fluid Lymphocyte Ratio

The ratio of serum LDH : pleural fluid lymphocyte was significantly higher in the malignant group. However, the sensitivity and specificity obtained from the ROC-derived cut-off level of >800 at best trade-off between them were 0.63 and 0.85, respectively. These were lower than the “cancer ratio” and “cancer ratio plus.” Further, the AUC of 0.68 suggests that this test would not be useful in clinical practice.

Thus “cancer ratio” and “cancer ratio plus” were found to be accurate in identifying MPE. When compared with the sensitivity and specificity of more advanced test such as tumour markers like CEA, CA15-3, CA125, and cyfra 21-1 in pleural fluid, the sensitivity and specificity of “cancer ratio” and “cancer ratio plus” were higher than these tests. The reported sensitivity and specificity of CEA, CA15-3, CA 125, and cyfra 21-1 were 0.65 and 0.97, 0.57 and 0.90, 0.68 and 0.83, and 0.53 and 0.79, respectively [9].

The strengths of this study include prospective data collection and consistency with previous reports. Our study has several limitations: first this was a single-centre observational study with a small cohort size, and with any study of this design there is the potential for confounding variables. Second, in some patients cell count was not reported due to degeneration of cells requiring exclusion of these patients. It is not possible to calculate the cancer ratio plus in such patients and this may pose a limitation to its use in clinical practice. Third, we did not study the other causes of lymphocytic exudative effusions such as connective tissue diseases, chylothorax, and pulmonary embolism to validate these results in this group of patients. Third, most patients with malignant effusion had lung cancer. This necessitates validation of our findings in extrapulmonary malignancies causing MPE. Since lymphoma related malignant pleural effusion can also have high ADA level and can mimic TPE, further study including larger number of patients with MPE from lymphoma is needed. However, lymphoma related MPE are rare as compared to the incidence of TPE and other causes of MPE especially in Asian countries. From the point of view of aetiology of MPE, in male patients, lung cancer is the most common cause and, in females, breast cancer is the most common cause, whereas the incidence of lymphoma causing pleural effusion is relatively lower. Fourth, none of the patients in our cohort of TPE had HIV. In HIV-positive patients with TPE, the percentage of lymphocytes may not be high which may affect the values of “cancer ratio plus.”

In conclusion, our “cancer ratio” maintained its specificity in diagnosing MPE in our prospective cohort, validating previous findings. In addition, the ratio of cancer ratio and pleural lymphocyte count, that is, “cancer ratio plus,” further increased the specificity of cancer ratio in identifying malignant pleural effusion. Thus “cancer ratio” and “cancer ratio plus” are the markers that can be derived just from routinely and universally performed biochemical tests but which can prompt the malignant nature of pleural effusion (especially in whom cytology is negative) with high accuracy without any additional test, cost, effort, or time. Such a screen can guide physicians in selecting out patients in whom to look for malignancy more actively as compared to taking watchful waiting approach or starting TB treatment empirically.

## Figures and Tables

**Table 1:** General characteristics and univariate analysis.

Variable	Total (*N* = 118)	Malignant pleural effusion (*N* = 84)	Tubercular pleural effusion (*N* = 34)	*P* value
Age	65 (19–87)	69 (35–87)	56 (19–87)	0.23
Pleural ADA (U/L)	10.6 (5–54)	9 (5–42)	42 (5–54)	0.001
Serum LDH (IU/L)	512 (322–2992)	525 (322–2992)	494 (336–947)	0.08
Pleural fluid lymphocyte count (%)	0.7 (0.1–1.0)	0.61 (0.10–1.0)	0.86 (0.60–1.0)	0.007
Cancer ratio	51.5 (7–173)	74 (15–173)	13 (7–67)	0.008
Serum LDH/pleural fluid lymphocyte count	765.5 (336–7771)	1015 (498–7771)	593 (336–1230)	0.006
Cancer ratio/pleural fluid lymphocyte count	87.2 (7.5–1295.2)	127 (29–1295)	16 (8–67)	0.002

Data presented in median (range).

**Table 2:** Logistic regression analysis for prediction of malignancy.

Variable	Coefficient	Odds	*P* value
Pleural ADA	−0.6011	0.54 (0.27–1.08)	0.0861
Serum LDH	0.0484	1.04 (0.99–1.11)	0.1015
Pleural fluid lymphocyte count	−10.224	0	0.1211
Cancer ratio	1.5744	0.20 (0.05–0.78)	0.0209
Serum LDH/pleural fluid lymphocyte count	0.0413	0.95 (0.92–0.99)	0.0474
Cancer ratio/pleural fluid lymphocyte count	1.6536	5.22 (1.35–20.14)	0.0163

**Table 3:** Cut-off for cancer ratio (serum LDH : pleural ADA ratio).

Cut-off level	Sensitivity (95% CI)	Specificity (95% CI)	PPV (95% CI)	NPV (95% CI)	PLR (95% CI)	NLR (95% CI)
>10	0.97 (0.90–0.99)	0.26 (0.13–0.44)	0.76 (0.67–0.84)	0.81 (0.47–0.96)	3.2 (2.2–4.6)	0.22 (0.06–0.80)
>20	0.95 (0.87–0.98)	0.85 (0.68–0.94)	0.94 (0.86–0.97)	0.87 (0.70–0.96)	16 (6.8–37.5)	0.13 (0.05–0.34)
>30	0.89 (0.80–0.94)	0.94 (0.78–0.98)	0.97 (0.90–0.99)	0.78 (0.61–0.88)	37.5 (9.5–147.3)	0.28 (0.15–0.50)
>40	0.76 (0.65–0.84)	0.94 (0.78–0.98)	0.96 (0.88–0.99)	0.61 (0.47–0.74)	32 (8.1–125.3)	0.62 (0.43–0.90)
>50	0.66 (0.55–0.76)	0.94 (0.78–0.98)	0.96 (0.87–0.99)	0.53 (0.40–0.66)	28 (7.1–109.3)	0.87 (0.64–1.18)
>60	0.57 (0.45–0.67)	0.97 (0.82–0.99)	0.97 (0.87–0.99)	0.47 (0.35–0.60)	48 (6.8–334.1)	1.09 (0.84–1.41)

**Table 4:** Cut-off for cancer ratio plus (cancer ratio: pleural fluid lymphocyte count).

Cut-off level	Sensitivity (95% CI)	Specificity (95% CI)	PPV (95% CI)	NPV (95% CI)	PLR (95% CI)	NLR (95% CI)
>20	1.0 (0.94–1.0)	64.7 (0.46–0.79)	0.87 (0.78–0.93)	1.0 (0.81–1.0)	7.0 (4.1–11.9)	0
>30	97.6 (0.90–0.99)	94.1 (0.78–0.98)	0.97 (0.90–0.99)	0.94 (0.78–0.98)	41 (10.4–161.3)	0.06 (0.01–0.2)
>40	92.8 (0.84–0.97)	94.1 (0.78–0.98)	0.97 (0.90–0.99)	0.84 (0.68–0.93)	39 (9.9–153.3)	0.18 (0.08–0.39)
>50	89.2 (0.80–0.94)	94.1 (0.78–0.98)	0.97 (0.90–0.99)	0.78 (0.61–0.88)	37.5 (9.5–147.3)	0.28 (0.15–0.50)
>60	82.1 (0.71–0.89)	97.0 (0.82–0.99)	0.98 (0.91–0.99)	0.68 (0.53–0.80)	69 (9.8–483)	0.45 (0.29–0.70)
